# Effect of admission time on mortality in an intensive care unit in Mainland China: a propensity score matching analysis

**DOI:** 10.1186/cc13053

**Published:** 2013-10-10

**Authors:** Min-Jie Ju, Guo-Wei Tu, Yan Han, Hong-Yu He, Yi-Zhou He, Hai-Lei Mao, Zhao-Guang Wu, Yi-Qing Yin, Jian-Feng Luo, Du-Ming Zhu, Zhe Luo, Zhang-Gang Xue

**Affiliations:** 1 Department of Anesthesiology and Surgical Intensive Critical Unit, Zhongshan Hospital, Fudan University, 136 Yi Xue Yuan Road, Shanghai 200032, People’s Republic of China; 2 Department of General Medical Practice, Zhongshan Hospital, Fudan University, 136 Yi Xue Yuan Road, Shanghai 200032, People’s Republic of China; 3 Department of General Surgery, Zhongshan Hospital, Fudan University, 136 Yi Xue Yuan Road, Shanghai 200032 People’s Republic of China; 4 Computer and Network Center, Zhongshan Hospital, Fudan University, 136 Yi Xue Yuan Road, Shanghai 200032, People’s Republic of China; 5 Department of Health Statistics and Social Medicine, School of Public Health, Fudan University, 138 Yi Xue Yuan Road, Shanghai 200032, People’s Republic of China

## Abstract

**Introduction:**

The relationship between admission time and intensive care unit (ICU) mortality is inconclusive and influenced by various factors. This study aims to estimate the effect of admission time on ICU outcomes in a tertiary teaching hospital in China by propensity score matching (PSM) and stratified analysis.

**Methods:**

A total of 2,891 consecutive patients were enrolled in this study from 1 January 2009 to 29 December 2011. Multivariate logistic regression and survival analysis were performed in this retrospective study. PSM and stratified analysis were applied for confounding factors, such as Acute Physiology and Chronic Health Evaluation II (APACHE II) score and admission types.

**Results:**

Compared with office hour subgroup (*n* = 2,716), nighttime (NT, *n* = 175) subgroup had higher APACHE II scores (14 vs. 8, *P* < 0.001), prolonged length of stay in the ICU (42 vs. 24 h, *P* = 0.011), and higher percentages of medical (8.6% vs. 3.3%, *P* < 0.001) and emergency (59.4% vs. 12.2%, *P* < 0.001) patients. Moreover, NT admissions were related to higher ICU mortality [odds ratio (OR), 1.725 (95% CI 1.118–2.744), *P* = 0.01] and elevated mortality risk at 28 days [14.3% vs. 3.2%; OR, 1.920 (95% CI 1.171–3.150), *P* = 0.01]. PSM showed that admission time remained related to ICU outcome (*P* = 0.045) and mortality risk at 28 days [OR, 2.187 (95% CI 1.119–4.271), *P* = 0.022]. However, no mortality difference was found between weekend and workday admissions (*P* = 0.849), even if weekend admissions were more related to higher APACHE II scores compared with workday admissions.

**Conclusions:**

NT admission was associated with poor ICU outcomes. This finding may be related to shortage of onsite intensivists and qualified residents during NT. The current staffing model and training system should be improved in the future.

## Introduction

Patients can become critically ill at any time of the day [1]. Ideally, critical care services should be organized to ensure optimal treatment availability to all patients on a 24/7 basis. In practice, however, the availability and quality of personnel and technology are often different between office hours (OH) and nighttime (NT) hours. The initial treatment is critical [2,3], and outcomes after intensive care admission partially depend on the time of patient admission. Bracco *et al*. [4] demonstrated the importance of early and accurate decision-making and the need for prompt patient supervision by an experienced specialist to avoid planning and execution mishaps.

We hypothesized that patients admitted to ICUs during NT may have worse outcomes. Investigators have evaluated this hypothesis in several studies, but the results of these studies were not uniform. Some studies showed an increased ratio of deaths among patients admitted during NT [5-7], whereas others documented a no-risk effect [8-12], or even a surprisingly protective effect [13,14], of NT admission relative to mortality. These studies were performed in Western countries where critical care medicine (CCM) is well-developed. However, this documentation is not available in developing countries with limited healthcare resources [15].

Evidence is mixed regarding the improved outcomes, including reductions in mortality and length of stay in the hospital (LOS_HOS_) or ICU (LOS_ICU_), for ICU patients whose care is directed by intensivists. Researchers have demonstrated in a number of studies that units with high-intensity physician staffing are associated with reduced mortality compared with units with low-intensity physician staffing [16]. However, a recent, large, retrospective study found higher odds of deaths for critically ill patients treated by critical care physicians compared with those treated by regular physicians [17]. This issue, especially in the context of NT admission, has not been resolved.

Our retrospective study was conducted in a university-affiliated, tertiary teaching hospital in Shanghai, People’s Republic of China, to evaluate the effect of admission time on ICU outcomes. We evaluated the Chinese mainland healthcare system and the relationship between admission time and patient outcome. A finding of worse outcomes of patients admitted during NT would affect intensivist staffing and administrators involved in both healthcare and insurance policy-making.

## Materials and methods

### Patient data

A total of 2,891 consecutive patients admitted to the general ICU of Zhongshan Hospital from 1 January 2009 to 29 December 2011 were enrolled in the study. The decision regarding their discharge from the ICU was based on our own transferring protocol. All patients signed the informed consent form. This study was approved by the Review Board of the Ethics Committee of Zhongshan Hospital, Fudan University, and was in compliance with the institution’s requirements.

The following clinical, physiological and outcome data were collected for evaluation: patient age, patient gender, primary diagnosis, Acute Physiology and Chronic Health Evaluation II (APACHE II) score at the time of admission, LOS_HOS_, LOS_ICU_, type of admission (surgical or medical, emergency or nonemergency), with or without transfusion during ICU stay and the need for mechanical ventilation.

### Staffing model

OH and NT were defined as 7:30 AM to 5:30 PM and 5:30 PM to 7:30 AM, respectively. We designated our staffing model as “10/7 on-site and NT on-call”. This model states that at least one attending intensivist is available in the ICU during OH for the entire week and formulates a plan for night patients. OH intensivists maintain responsibility for all patients and are available by telephone to in-hospital residents of medicine, surgery or anesthesiology at night. Our department usually has three to four residents from surgery, anesthesiology or medicine every month. Most of these residents have taken the residency training programs of Shanghai for at least one year. The duration of the training programs is based on the resident’s educational background. Therefore, a resident with a doctoral degree should undergo a one-year training program, whereas a resident with a master’s degree should train for two years. However, the training courses are focused on residents’ specialties and lack integration of CCM training. Thus, basic CCM training is provided through a weekly Journal Club, morning course, daily rounds and clinical practice. This staffing model is maintained for the entire week, including weekdays (Monday to Friday) and weekends (Saturday and Sunday).

During the day and night and on weekdays and weekends, the nurse-to-patient ratio was 1:2.5. All nurses were certified in critical care. Other disciplines, such as radiology and ultrasound departments, were available around the clock.

Zhongshan Hospital has an effective consultation system of senior surgeons and physicians from other departments, such as anesthesia, cardiology and pulmonary medicine. We maintained horizontal relationships with these specialists who provided valuable advice during the day or at night and on weekdays or weekends. Multidisciplinary intensive care was also administered to our patients [18].

### Study design and statistics

A crude analysis of the 2,891 patients was performed, and mortality between OH and NT admissions was compared. The data were analyzed using SPSS 15.0 software (SPSS, Inc, Chicago, IL, USA) and tested for normal distribution using the Kolmogorov–Smirnov test. Continuous variables were expressed as the mean ± SD or as the median and full range if the assumption of a normal distribution was violated. Categorical variables were expressed as numeric values and percentages. Comparisons of continuous variables were performed using the Mann–Whitney *U* test and the Wilcoxon rank-sum test, and the *χ*^2^ test or Fisher’s exact test was applied for categorical variables. Multivariate logistic regression was used to assess mortality at 28 days. The survival curves were further estimated using the Kaplan–Meier method and compared by applying the logrank test. The Cox regression model was used to perform univariate and multivariate analyses. Propensity score matching (PSM) was carried out because of imbalance in baseline characteristics. A multivariable logistic regression model, including the variables of emergency admissions, surgical or medical patients and APACHE II scores, was developed to calculate the propensity score. The psmatch2 macro in Stata 11.0 software (StataCorp LP, College Station, TX, USA) was used for PSM. The PSM and analytical methods used in this study were based on several sources [19]. The propensity score represented the probable time when a patient was transported into the ICU, based on variables that were known or suspected to be confounding factors for ICU mortality. A *P* value less than 0.05 (two-tailed) was considered statistically significant.

## Results

### Basic clinical data

The distributions of major clinical data were analyzed first (Table 1). The mean age of the cohort was 61 ± 14 years, and 1,944 patients (67.2%) were male. Most patients (*n* = 2,786, 96.4%) were admitted postoperatively or underwent surgery during their ICU stay. The emergency admission rate of this cohort was 15.08% (*n* = 436). The median LOS in the hospital and in the ICU, as well as in the hospital before ICU admission, were 14 days, 24 hours and 3 days, respectively.

**Table 1:** **Distributions of major clinical data and study items**^**a**^

**Clinical data**		**All (*N*** **= 2,891)**	**Admission group**		
			**NT (*n*** **= 175)**	**OH (*n*** **= 2,716)**	***P***
Mean age (years)		61	62	61	0.515^b^
Gender (*n*)	Male	1,944	124 (71.9%)	1,820 (67.0%)	0.293^c^
	Female	947	51 (29.1%)	896 (33.0%)	
Emergency (*n*)	No	2,455	71 (40.6%)	2,384 (87.8%)	<0.001^c^
	Yes	436	104 (59.4%)	332 (12.2%)	
Source (*n*)	Medical	105	15 (8.6%)	90 (3.3%)	<0.001^c^
	Surgical	2,786	160 (91.4%)	2,626 (96.7%)	
Transfusion (*n*)	No	2,571	151 (86.3%)	2,420 (89.1%)	0.250^c^
	Yes	320	24 (13.7%)	296 (10.9%)	
Mechanical ventilation (*n*)	No	2,550	149 (85.1%)	2,401 (88.4%)	0.201^d^
	NIV	21	1 (0.6%)	20 (0.7%)	
	Intubation	246	22 (12.6%)	224 (8.2%)	
	Tracheotomy	74	3 (1.7%)	71 (2.7%)	
Median APACHE II score		8	14	8	<0.001^b^
ICU outcome (*n*)	Dead	125	27 (15.4%)	98 (3.6%)	<0.001^c^
	Alive	2,766	148 (74.6%)	2,618 (96.4%)	
Median LOS_HOS_ (days)		14	16	14	0.581^b^
Median LOS_ICU_ (hours)		24	42	24	0.011^b^
Mortality (28 days), n (%)		111 (3.8%)	25 (14.3%)	86 (3.2%)	<0.001^c^

^a^APACHE II, Acute Physiology and Chronic Health Evaluation II; LOS_HOS_, length of stay in hospital; LOS_ICU_, length of stay in ICU; NIV, noninvasive ventilation; NT, nighttime; OH, office hours. *P* values were derived from the comparison between the NT and OH groups. ^b^Mann–Whitney *U* test. ^c^χ^2^ test. ^d^Fisher’s exact test.

**Figure 1:**
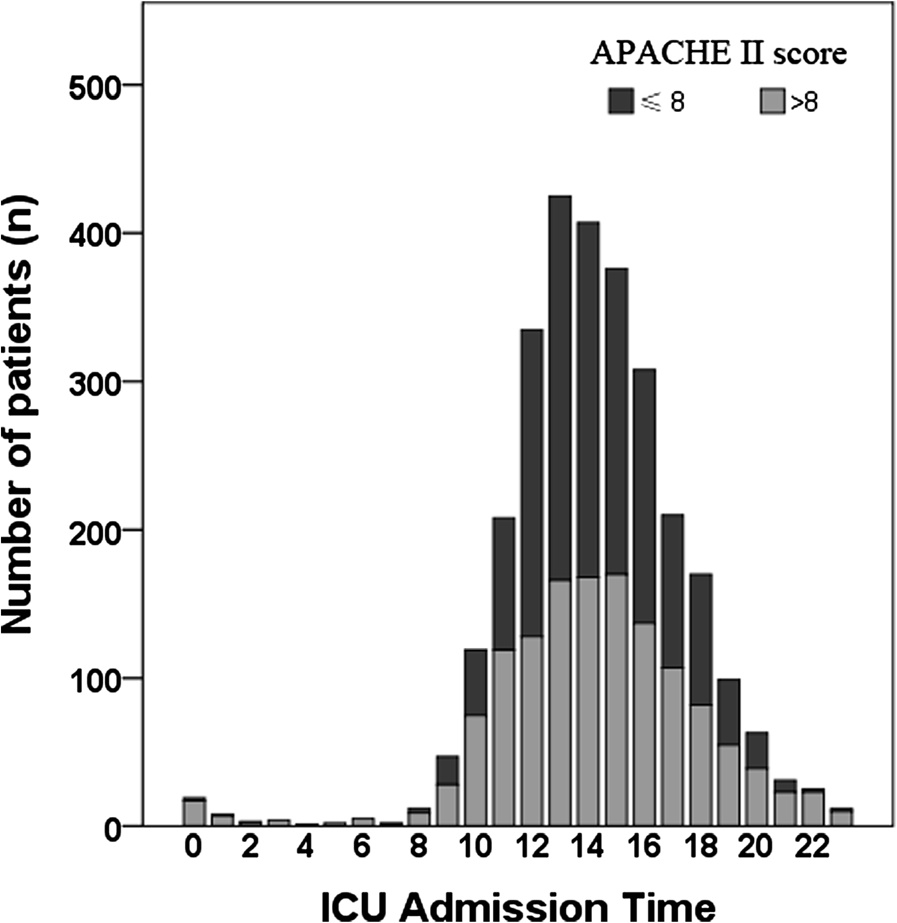
**Distribution of admissions per hour.** APACHE II, Acute Physiology and Chronic Health Evaluation II.

As the admission time was an important variable, we next analyzed the distribution of ICU admissions per hour, which is shown in Figure 1. Exactly 2,716 patients (93.9%) were admitted during OH, and 175 (6.1%) patients were NT admissions. The following comparisons between NT and OH admissions demonstrate that the ratios of emergency admissions (59.4% vs. 12.2%; *P* < 0.001), medical patients (8.6% vs. 3.3%; *P* < 0.001) and median APACHE II scores (14 vs. 8; *P* < 0.001) were significantly higher in the NT group than in the OH group (Table 1). In addition, the LOS_ICU_ of NT admissions was longer than that of OH admissions (median 42 vs. 24 hours; *P* = 0.011), though the LOS_HOS_ was not significantly different between them (median 16 days in NT group vs. 14 days in OH group; *P* = 0.581). We next assessed whether APACHE II score influenced the correlation between NT admission and LOS_ICU_. The groups were divided into high and low APACHE II score subgroups. Considering that the median APACHE II score for both total patients and OH patients was 8 (Table 1), we defined the high and low APACHE II score subgroups as APACHE II scores >8 and ≤8, respectively. In fact, the results showed that NT admission was associated with prolonged LOS_ICU_, regardless of APACHE II score subgroup (median high APACHE II score subgroup: 119 hours in NT vs. 90 hours in OH (*P* < 0.001), median low APACHE II score subgroup: 48 hours in NT vs. 38 hours in OH (*P* = 0.008)).

### Mortality at 28 days

To seek the primary variables that affect clinical outcomes, the correct analyses with mortality at 28 days were performed. The univariate analysis results showed that NT vs. OH admission time (14.3% vs. 3.2%; *P* < 0.001), emergency vs. nonemergency admission (17.2% vs. 1.5%; *P* < 0.001) and surgical vs. medical admission (3% vs. 25.7%; *P* < 0.001) were related to the 28-day mortality rate (Table 2). Next, we added these three factors, along with APACHE II scores, into the multivariate logistic regression analysis. This analysis also indicated that admission time was significantly related to the 28-day mortality rate (odds ratio (OR) = 1.920 (95% CI = 1.171 to 3.150); *P* = 0.01) (Table 3).

**Table 2:** **Univariate analysis of 28-day mortality**^**a**^

**Variables**		**Dead**	**Alive**	***P***
Admission time, *n* (%)	NT	25 (14.3)	150 (85.7)	<0.001^b^
	OH	86 (3.2)	2,630 (96.8)	
Emergency admission, *n* (%)	No	36 (1.5)	2,419 (98.5)	<0.001^b^
	Yes	75 (17.2)	361 (82.8)	
Source, *n* (%)	Medical	27 (25.7)	78 (74.3)	<0.001^c^
	Surgical	84 (3.0)	2,702 (97)	

^a^NT, nighttime; OH, office hours. ^b^χ^2^ test. ^c^Fisher’s exact test.

**Table 3:** **Multivariate analysis of 28-day mortality**^**a**^

			**95% CI**	
**Variables by cohort**	***P***	**Odds ratio**	**Lower**	**Upper**
All patients				
Admission time (NT vs. OH)	0.010	1.920	1.171	3.150
Emergency admission (yes vs. no)	<0.001	51.241	36.234	72.463
Source (medical vs. surgical)	0.959	1.014	0.606	1.697
APACHE II score	<0.001	1.032	1.017	1.047
PSM cohort				
Admission time (NT vs. OH)	0.022	2.187	1.119	4.271
Emergency admission (yes vs. no)	0.788	1.135	0.452	2.848
Source (medical vs. surgical)	0.071	0.404	0.151	1.079
APACHE II score	<0.001	1.161	1.103	1.222

^a^APACHE II, Acute Physiology and Chronic Health Evaluation II; CI, confidence interval; NT, nighttime; OH, office hours; PSM, propensity score matching. All *P* values were calculated using multivariate logistic regression.

### Survival analysis

#### Univariate and multivariate analyses in the entire cohort

**Figure 2:**
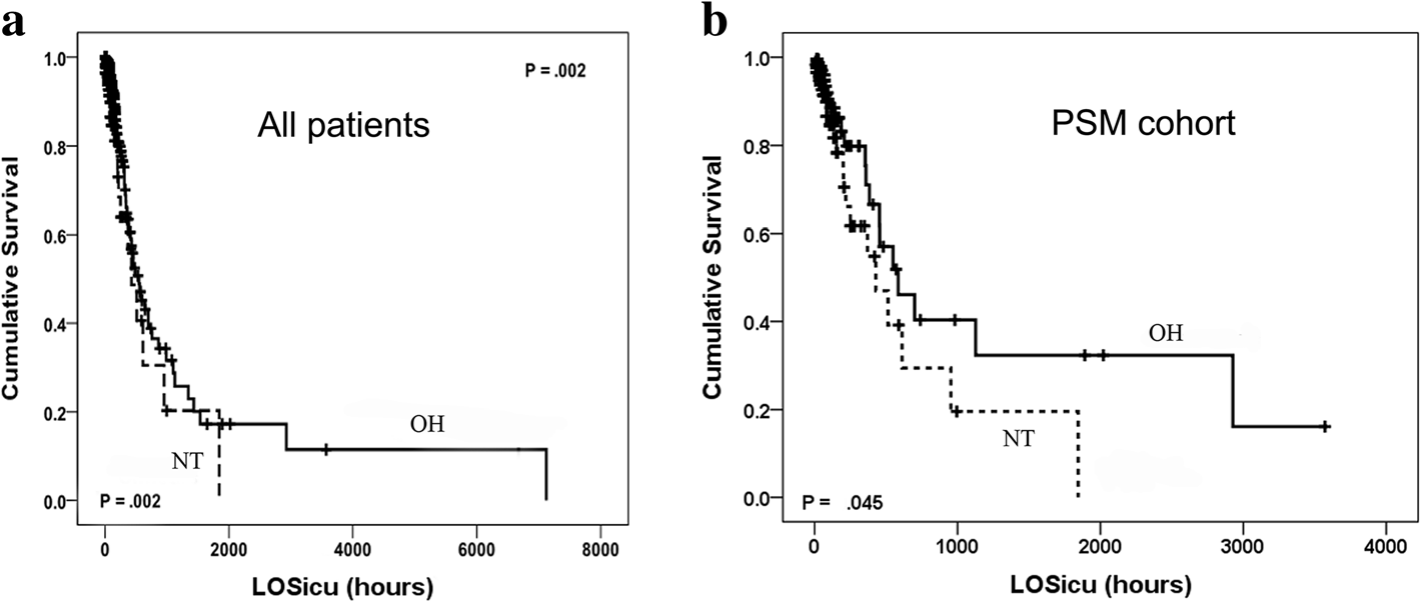
**Kaplan–Meier curves of survival differences among ICU patients.** ICU survival of office hours (OH) admissions was significantlyhigher than that of nighttime (NT) admissions **(a)**. Propensity score matching (PSM) analysis revealed that NT admissions corresponded to poorer ICU outcomes **(b)**. The dashed lines represented nighttime (NT) admissions, and the solid lines represented office hours (OH) admissions.

The univariate analysis showed that ICU mortality of NT admissions was significantly higher than that of OH admissions (15.4% vs. 3.6%; *P* = 0.002) (Figure 2a). To validate the independent correlation between ICU admission time and ICU outcome, a multivariate survival analysis was conducted, including all of the clinical variables (Table 4). Only NT vs. OH admission time (OR = 1.752, 95% CI = 1.118 to 2.744; *P* = 0.01) and high vs. low APACHE II score (OR = 1.113; *P* < 0.001) represented risk factors for ICU mortality.

**Table 4:** **Multivariate survival analysis**^**a**^

**Variables**	***P***	**OR**	**95% CI for HR**	
			**Lower**	**Upper**
Age (years)	0.199	1.008	0.996	1.021
Gender (female/male)	0.997	0.999	0.648	1.542
Source (medical/surgical)	0.153	1.408	0.880	2.251
Emergency admission (yes/no)	0.254	1.270	0.842	1.914
Transfusion (no/yes)	0.541	0.878	0.578	1.333
Mechanical ventilation (no/NIV/intubation/tracheotomy)	0.060	0.829	0.682	1.008
Admission day (weekday/weekend)	0.198	0.724	0.443	1.183
Admission time (NT/OH)	0.010	1.725	1.118	2.744
APACHE II score (>8/≤8)	<0.001	1.113	1.082	1.145

^a^APACHE II, Acute Physiology and Chronic Health Evaluation II; CI, confidence interval; HR, hazard ratio; NIV, noninvasive ventilation; NT, nighttime; OH, office hours; OR, odds ratio. All *P* values were calculated using the Cox proportional hazards regression model.

#### Stratified analyses

**Figure 3:**
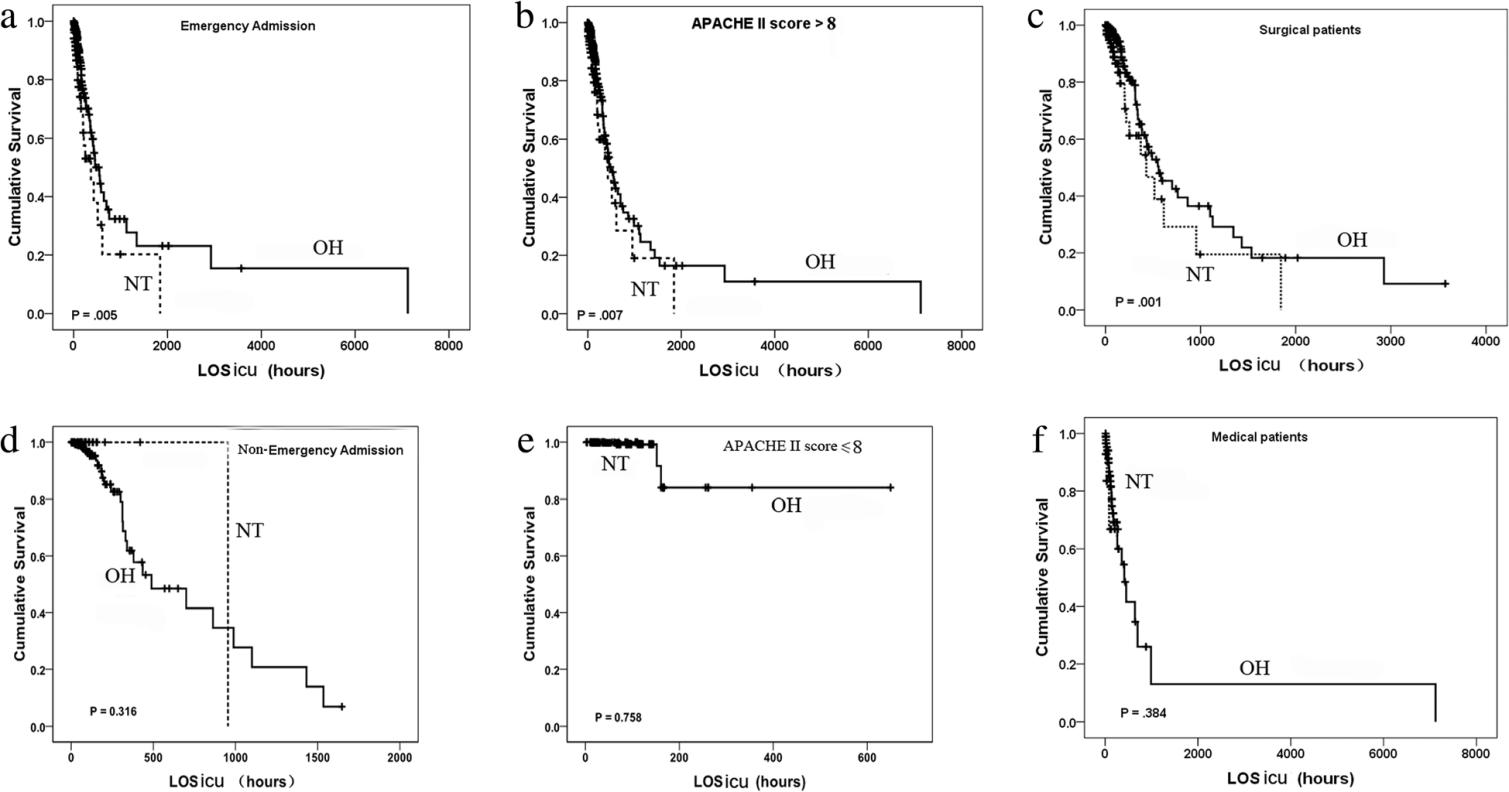
**Kaplan–Meier curves of ICU survival differences in stratified analysis.** In the subgroups of emergency admissions **(a)**, patients with greater Acute Physiology and Chronic Health Evaluation II (APACHE II) scores (>8) **(b)** and surgical patients **(c)**, mortality was significantly reduced during office hours (OH) compared with nighttime (NT). On the contrary, admission time did not correlate with ICU outcome in nonemergency admissions **(d)**, the subset with low APACHE II scores (≤8) **(e)** and medical patients **(f)**. LOS_icu_, length of stay in the ICU. The dashed lines represented nighttime (NT) admissions, and the solid lines represented office hours (OH) admissions.

Table 1 shows that factors including the distributions of admission types (emergency vs. nonemergency; surgical vs. medical) and APACHE II scores were significantly different between the NT and OH groups. The foregoing were confounding factors in the relationship between ICU mortality and admission time. To elucidate this issue, stratified analyses were performed, which showed that OH admissions were still related to reduced ICU mortality for emergency patients (OR = 0.518; *P* = 0.005) (Figure 3a), the elevated APACHE II score subgroup (OR = 0.558; *P* = 0.007) (Figure 3b) and surgical patients (OR = 0.462; *P* = 0.001) (Figure 3c). On the contrary, admission time did not correlate with ICU outcomes for nonemergency admissions, the low APACHE II score subgroup or medical patients (Figure 3d to 3f).

#### Propensity score matching analysis

The efficiency of stratified analyses was limited to some extent because of remarkable bias in patient distribution. Moreover, other potential confounding factors were identified. Thus, we applied a 1:1 PSM ratio to minimize these effects and included factors such as admission type (emergency vs. nonemergency, surgical vs. medical) and APACHE II score. Additional file 1: Table S1 lists the multiple logistic regression results in the PSM analysis. In the PSM analysis, we selected 175 patients from OH admissions as matched pairings for the 175 NT admissions. The main clinical characteristics were balanced and evenly distributed between these two types of patients (Table 5). We found that NT admissions were related to lower ICU survival (Figure 2b) and higher mortality at 28 days (OR = 2.187, 95% CI = 1.119 to 4.271; *P* = 0.022) (Table 3). We also carried out a 1:2 match for the PSM analysis and obtained similar results, and admission time was proved again to be related to mortality at 28 days (Additional file 1: Figure S1 and Tables S2 and S3).

**Table 5:** **Demographic and clinical characteristics of the propensity score matching population**^**a**^

		**OH**		**NT**		
		**Total (dead)**	**Median LOS**_**ICU**_**(hours)**	**Total (dead)**	**Median LOS**_**ICU**_**(hours)**	***P***
APACHE II score (median)		14 (23)	65	14 (27)	42	0.983^a^
Emergency admission (*n*)	No	71 (2)	24	71 (1)	38	1.0^b^
	Yes	104 (21)	90	104 (26)	58	
Source (*n*)	Medical	15 (5)	95	15 (3)	37	1.0^b^
	Surgical	160 (18)	48	160 (24)	43	
Mean age (years)		65 (23)	65	62 (27)	42	0.139^a^
Gender (*n*)	Male	115 (16)	66	124 (22)	60	0.301^b^
	Female	60 (7)	49	51 (5)	37	
Mechanical ventilation (*n*)	No	145 (6)	43	149 (8)	37	0.840^c^
	NIV	2 (0)	145	1 (0)	59	
	Intubation	23 (14)	87	22 (18)	58	
	Tracheotomy	5 (3)	307	3 (1)	200	
Transfusion (*n*)	No	147 (13)	46	151 (12)	38	0.548^b^
	Yes	28 (10)	178	24 (15)	59	

^a^APACHE II, Acute Physiology and Chronic Health Evaluation II; LOS_HOS_, length of stay in hospital; LOS_ICU_, length of stay in ICU; NIV, noninvasive ventilation; NT, nighttime; OH, office hours. ^a^Mann–Whitney *U* test. ^b^χ^2^ test. ^c^Fisher’s exact test.

#### Weekend admission

**Figure 4:**
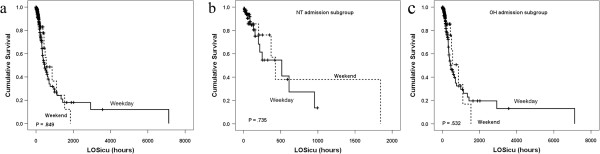
**Survival analyses for weekend and weekday admissions.** No statistically significant difference was found between weekday and weekend admissions **(a)**. Mortality rates between weekend and workday admissions were similar in the subgroups of nighttime (NT) **(b)** and office hours (OH) **(c)** admissions. LOS_icu_, length of stay in the ICU. The dashed lines represented weekend admissions, and the solid lines represented weekday admissions.

The APACHE II scores were higher among weekend admissions compared with weekday admissions (*P* < 0.001) (Additional file 1: Table S4). This finding should have resulted in a difference in mortality, but no significant mortality difference was observed between weekend and weekday admissions (*P* = 0.849) (Figure 4a). We also performed analysis of admission times (NT and OH) to clarify these paradoxical results. In the NT subgroup, the APACHE II scores between weekday and weekend admissions were comparable (Additional file 1: Table S4), and no difference in mortality was observed (Figure 4b). By contrast, in the OH group, the APACHE II scores were significantly higher during weekends than on weekdays (*P* < 0.001) (Additional file 1: Table S4), but ICU mortality was similar (Figure 4c).

## Discussion

### Different patient outcomes between office hours and nighttime admissions

Several publications have identified differences in mortality between patients admitted during OH and NT [5,6,8,9,12,20]. In our present study, we found that ICU mortality, 28-day mortality and LOS_ICU_ of patients admitted during NT were significantly higher than those of patients admitted during OH, especially for emergency patients, high APACHE II score patients and surgical patients. However, the definition of OH is not uniform yet. In particular, the level of support systems available in each study was not clear [21].

In the present study, we defined OH as hours when a qualified intensivist was available on-site for patient care, as described in the report by Cavallazzi *et al*. [22]. NT coverage in our ICU was provided by non-CCM residents only, and one intensivist was on-call at home. In the context of ICU mortality, confounding factors such as disease severity (APACHE II score), patient age and surgical vs. nonsurgical patients should be considered. To clarify this confounding bias, we first applied multivariate survival analyses and logistic regression including these confounding factors. Furthermore, we performed PSM analysis (at 1:1 and 1:2 ratios). Our results show that ICU mortality and 28-day mortality of NT admissions remained higher than those of OH admissions. Consequently, our results attest to the correlation between NT admissions and poor clinical outcomes.

### Higher mortality in nighttime admissions might be related to staffing model

Considering the apparent differences in staffing between NT and OH, we proposed that the effect of NT admission on mortality might be related to the staffing arrangement pattern. Previous findings were helpful in substantiating this notion. First, preventable adverse events were more likely to occur during NT, when house officers were less likely to receive supervision [23]. Second, prompt diagnosis and initiation of specific therapies by an intensivist were also crucial at the time of the critically ill patient’s admission [4], whereas delayed treatment, such as early goal-directed therapy, was less effective [24,25]. Third, increased NT intensivist or nurse staffing yielded lower hospital mortality and adverse events [26,27].

Our comparison between weekend and weekday admissions also provides potent support for this notion. Despite the fact the APACHE II scores were higher and were significantly associated with a higher rate of ICU mortality in weekend patients, no difference in mortality between weekend and weekday admissions was observed. Moreover, our results show that APACHE II scores were significantly higher during weekends than on weekdays but not in the NT subgroup. This means that the higher APACHE II scores of weekend patients is attributable to the admission of more critical patients during OH than NT. Therefore, APACHE II score may be not the most dominant factor related to ICU mortality in the analysis. Considering our staffing model, on-site intensivists covered and managed patients during OH on both weekdays and weekends, but not during NT on either weekdays or weekends. These data indicate that the staffing model may be the relevant factor underlying the nonsignificant increase in the mortality of patients with elevated APACHE II scores admitted on weekends (especially during OH). Hence, higher mortality at night may be related to the difference in staffing coverage (intensivists on-site vs. on-call).

Moreover, we found that a higher likelihood of patient admission with elevated APACHE II score occurred during NT than OH (134/175 vs. 1244/2716, *P* < 0.001). APACHE II score was also independently related to mortality. Proper interventions for these severely ill patients during NT are critical to their outcome. However, the shortage of well-trained ICU residents and onsite intensivist supervision may restrict the treatment of patients admitted during NT.

### ICU training for residents may be a promising choice in China

The presence of 24-hour on-site intensivists is ideal [1], but this coverage requires more personnel. The existing and projected shortfalls in the intensivist workforce, the critical care–oriented training system for residents and financial support, especially in developing countries [15,28], may preclude the widespread adoption of this model [29]. In China, the lack of financial resources has also led to the impossibility of adopting the 24/7 on-site staffing model in the near future. The model proposed by the European Society of Intensive Care Medicine states that dedicated residents could provide off-hours coverage with daily attending intensivist supervision [30], which is feasible and provides another choice for developing countries.

Our current staffing model is similar to that proposed in the latest study by Kerlin *et al*. [31]. However, they found no improvement in ICU mortality when they applied on-site NT intensivist staffing. The main difference between our study and that of Kerlin *et al*. is the ICU training for residents. Their residents were well-trained; bedside intensivists may not add to the quality of care provided by residents [31]. In contrast, our residents received few ICU training courses. Handling critically ill patients properly without onsite intensivists is difficult for them. To facilitate the increased number of needed residents, providing our non-CCM residents with sufficient training and upgrading our clinical approaches and protocols are of primary importance.

### Limitations and perspectives of the study

We identified three major limitations of this study. First, our study was retrospective, with many confounding factors and selection bias. In particular, our study population comprised high percentages of OH admissions (93.9%) and surgical patients (96.4%). The reasons for this distribution are as follows. (1) Although we have a mixed ICU, most patients were admitted from the operating room or postoperative recovery room (*n* = 2,786). Most patients were admitted after surgery during the day, resulting in fewer available ICU beds during NT. (2) The difference in NT definition resulted in different NT admission rates to some extent. We realized that unbalanced distribution might influence the results. (3) According to our stratified analyses, admission time was related to ICU mortality among surgical patients (Figure 3c) but not among medical patients (Figure 3f). (4) We conducted a single-center study within one ICU in a large tertiary referral center. (5) Our study was carried out in mainland China. The results of this study thus may not be generalizable to all ICUs, and further prospective randomized controlled trials should be performed for comparison with our results.

To the best of our knowledge, no other studies have previously been carried out in China regarding the outcomes of ICU patients admitted at night. Given the current financial and staffing situations in China, establishing a CCM training system and improving clinical protocols should be carried out so that dedicated trainees can accomplish NT coverage with on-call attending intensivist supervision. Reduced ICU mortality constitutes only a small part of quality care [32]. Thus, further studies should explore the relationship between admission time and quality of other aspects of ICU care, such as unplanned extubation ratio and length of mechanical ventilation.

## Conclusions

Our study indicates that the higher mortality rate among NT admissions may be related to differences in staffing coverage. Despite limited staffing and financial resources in developing countries, improving the current ICU staffing model and optimizing the clinical protocol for non-CCM residents are necessary.

## Key messages

• NT admission is related to ICU mortality even after PSM analysis.

• Comparisons between weekend and weekday admissions supported our notion that higher mortality at night may be related to differences in staffing coverage.

• Given the current shortage of financial and staffing resources, as well as the insufficient ICU training courses for residents, upgrading our CCM training system and improving our clinical protocols so that dedicated trainees can accomplish NT coverage with on-call intensivist supervision is a primary necessity.

## Abbreviations

APACHE II: Acute Physiology and Chronic Health Evaluation II; CCM: Critical care medicine; LOSHOS: Length of stay in the hospital; LOSICU: Length of stay in the ICU; NT: Nighttime; OH: Office hours; PSM: Propensity score matching.

## Competing interests

The authors have no conflicts of interest to declare.

## Authors’ contributions

MJJ, GWT, YH, HYH, YZH, YQY, HLM and ZL were involved with the study design, data collection, data analysis and manuscript preparation. MJJ, JFL, ZGW, ZGX and DMZ were involved in the statistical design and manuscript preparation. All authors read and approved the final manuscript.

## Supplementary Material

Additional file 1: Table S1 Multiple logistic regression results in 1:1 PSM. **Table S2.** Distributions of variables before and after 1:2 PSM. **Table S3.** Multivariate logistic regression for 28-day mortality of 1:2 PSM. **Table S4.** Distribution of APACHE II scores among different admission combinations. **Figure S1.** Propensity score histogram for 1:2 PSM. Click here for file
