# Supplementary material for: Effect of admission time on mortality in an intensive care unit in Mainland China: a propensity score matching analysis
Source: Crit Care. 2013 Oct 10;17(5):R230. doi: 10.1186/cc13053 (PMC4055975; doi:10.1186/cc13053)
Supplement: Additional file 1: Table S1 — Multiple logistic regression results in 1:1 PSM. Table S2. Distributions of variables before and after 1:2 PSM. Table S3. Multivariate logistic regression for 28-day mortality of 1:2 PSM. Table S4. Distribution of APACHE II scores among different admission combinations. Figure S1. Propensity score histogram for 1:2 PSM. [file cc13053-S1.doc]

**Appendix**

**Table 1 Multiple logistic regression results in 1:1 PSM**

|  | Odds Ratio | Z | P>|z| | 95% CI | |
| --- | --- | --- | --- | --- | --- |
|  |  |  |  | Lower | Upper |
| Source (Surgical/ Medical) | 1.612345 | 1.54 | 0.124 | 0.8766 | 2.9655 |
| Emergency (Yes/No) | 8.038943 | 10.86 | 0 | 5.519 | 11.7093 |
| APACHE II score | 1.048269 | 3.83 | 0 | 1.0232 | 1.0739 |

**Table 2. Distributions of variables before and after 1:2** PSM

| Variable | Sample^*^ | Mean | | *t*-test | |
| --- | --- | --- | --- | --- | --- |
|  |  | Treated | Control | *t* | *P* |
| Source | Unmatched | 0.91429 | 0.96686 | −3.61 | <0.001 |
|  | Matched | 0.91429 | 0.90286 | 0.37 | 0.712 |
| Emergency | Unmatched | 0.59429 | 0.12224 | 17.81 | <0.001 |
|  | Matched | 0.59429 | 0.56571 | 0.54 | 0.589 |
| Transfusion | Unmatched | 0.30286 | 0.10898 | 7.71 | <0.001 |
|  | Matched | 0.30286 | 0.31429 | −0.21 | 0.818 |
| APACHE II score | Unmatched | 14.383 | 9.2883 | 11.77 | <0.001 |
|  | Matched | 14.383 | 14.834 | −0.57 | 0.57 |

*****Unmatched, before PSM; Matched, after PSM

**Table 3. Multivariate logistic regression for 28-day mortality**

**of 1:2 PSM**

|  | *P* | OR | 95% CI | |
| --- | --- | --- | --- | --- |
|  |  |  | Lower | Upper |
| Admission time (NT vs. OH) | 0.043 | 2.024 | 1.023 | 4.004 |
| Emergency admission (Yes vs. No) | 0.791 | 1.107 | 0.521 | 2.351 |
| Source (medical vs. surgical) | 0.063 | 0.410 | 0.160 | 1.049 |
| APACHE II score | <0.001 | 1.184 | 1.120 | 1.252 |

NT, nighttime; OH, office hours

**Table 4** Distribution of APACHE II scores among different admission combinations

|  |  | All Patients | | | NT Admission | | | OH Admission | | |
| --- | --- | --- | --- | --- | --- | --- | --- | --- | --- | --- |
|  |  | Work- day | Weekend | *P* | Work- day | Weekend | *P* | Work- day | Weekend | *P* |
| APACHE II score | mean | 9 | 14 | < 0.001^*^ | 13.8 | 16.5 | **0.051**^*^ | 9 | 13.9 | <0.001^*^ |
|  | >8 (n) | 1,244 | 134 | <0.001^†^ | 103 | 31 | 0.243^†^ | 1,141 | 103 | <0.001^†^ |
|  | ≤8 (n) | 1,472 | 41 |  | 35 | 6 |  | 1,437 | 35 |  |

*Mann-Whitney U test; †chi-square test.

NT, nighttime; OH, office hours; APACHE II, Acute Physiologic and Chronic Health Evaluation II.

**Fig 1.** **Propensity score histogram for 1:2 PSM**
